# Accurate Stress Analysis of Variable Angle Tow Shells by High-Order Equivalent-Single-Layer and Layer-Wise Finite Element Models

**DOI:** 10.3390/ma14216486

**Published:** 2021-10-28

**Authors:** Alberto Racionero Sánchez-Majano, Rodolfo Azzara, Alfonso Pagani, Erasmo Carrera

**Affiliations:** 1 Department of Mechanical and Aerospace Engineering, Politecnico di Torino, 10129 Turin, Italy; alberto.racionero@polito.it (A.R.S.-M.); rodolfo.azzara@polito.it (R.A.); erasmo.carrera@polito.it (E.C.); 2 Department of Mechanical Engineering, College of Engineering, Prince Mohammad Bin Fahd University, P.O. Box 1664, Al Khobar 31952, Saudi Arabia

**Keywords:** composite aerospace structures, stress analysis, numerical methods

## Abstract

New concepts of lightweight components are conceived nowadays thanks to the advances in the manufacture of composite structures. For instance, mature technologies such as Automatic Fibre Placement (AFP) are employed in the fabrication of structural parts where fibres are steered along curvilinear paths, namely variable angle tow (VAT), which can enhance the mechanical performance and alleviate the structural weight. This is of utmost importance in the aerospace field, where weight savings are one of the main goals. For that reason, shell structures are commonly found in the aerospace industry because of their capabilities of supporting external loadings. Straight-fibre composite shell structures have been studied in recent decades and, now, spatially varying composite shells are attracting the attention of manufacturers. This work analyses the mechanical behaviour of VAT composite shells subjected to different external loadings and boundary conditions. The Carrera Unified Formulation (CUF) is employed to obtain the different structural models in a systematic and hierarchic manner. The outcomes of such numerical models are discussed and compared with commercial software Abaqus.

## 1. Introduction

Nowadays, an increasing number of engineering applications, such as civil, marine, automotive and aerospace, are employing thin-walled structures—as an example, bridges and oil rings, ships, chassis and aircraft, respectively. Specifically, shells consist of curved lightweight constructions, which became very widespread in structural engineering thanks to their high performance when supporting external loads. Such outstanding mechanical properties stem from the coupling between the membrane and flexural behaviour, induced by the curvature. The geometric characteristics of shell structures, including the initial curvatures, have a direct influence on the stiffness properties [1]. Therefore, a proper design of such structures is crucial to perform accurate stress predictions under different loading and boundary conditions. The popularity of shell models is thanks to their lower computation cost compared to three-dimensional (3D) models.

As stated by Kapania [2], composite shell structures are playing a crucial role in several branches of engineering. Particularly in aerospace, composite materials offer an attractive possibility to more traditional types of construction due to their corrosion resistance, high strength-to-weight ratio, ease of formability, excellent fatigue resistance and tailoring ability. In fact, the stiffness, strength and flexibility characteristics can be controlled in different directions allowing one to obtain a significant increase in performance. Furthermore, the possibility of applying fibres that are not constrained along straight paths but can follow curvilinear paths leads to numerous advantages from the point of view of structural efficiency, increasing the design space and improving the tailoring process [3,4,5]. These new advanced composite structures, called variable angle tow (VAT), have attracted a lot of interest because they allow the manufacturing of variable stiffness composite laminates (VSCL) without discontinuity in the material while maximising the ratio of stiffness to mass.

The VAT methodology is not new, but it has recently received renewed interest due to significant technological improvements in the automatic manufacturing process. Essentially, the advanced Automated Fibre Placement (AFP) technique [6] and the Continuous Tow Shearing (CTS) process [7] allow the fibre orientation angle of a layer to vary with respect to one or more spatial directions. The literature concerning the VAT theories is vast. As an example, several progressive damages and failure analyses on classical and VAT composite panels were performed by Lopes et al. [8], showing the potentialities of the variable stiffness structures to redirect the load fluxes to the stiffer edge area to improve the structural performance. Curvilinear fibres were adopted by Hyer and Lee [9] to change the stress concentration around a hole. Stodieck et al. [10] investigated the possibility of improving the aeroelastic tailoring of a rectangular unswept laminate wing using the VAT methodology with the comparison with the classical one. Setoodeh et al. [11] adopted the VAT technique to maximise the buckling load of composite panels.

Over the years, researchers and scientists have developed several efficient shell theories. For example, the studies of Poisson, [12], Love [13], Mindlin [14], Kirchhoff [15], Reissner [16] and Cauchy [17] represent the classical formulation of the shell models. Typically, these classical theories are adopted in the commercial codes. However, the applicability of classical theories is limited to a narrow range of applications, for example, when dealing with the thin-walled structure and without local effects. On the contrary, more accurate shell formulations are needed when transverse stresses analyses are required. Recently, several higher-order 2D formulations were formulated to improve the accuracy of classical theories. For example, Reddy [18] provided a simple high-order theory for laminated composite two-dimensional (2D) structures. A shear model considering a parabolic distribution of transverse shear deformations in the thickness direction was presented by Reddy and Liu [19]. An assessment of the relevance of displacement variables in refined theories for isotropic and multilayered shells using an axiomatic/asymptotic technique was carried out by Mashat et al. [20]. Carrera [21,22] developed several refined shell theories in the framework of the Carrera Unified Formulation (CUF). Cinefra and Carrera [23] performed linear analyses of composite cylindrical structures using finite shell elements with different through-the-thickness kinematics. A useful review of methods and guidelines for the choice of the shell model was reported by Petrolo and Carrera [24]. A detailed review of the theories is not within the scope of this work. Readers are referred to [25,26] for other significant works on refined shell formulations.

There exists a limited number of works regarding the stress evaluation of VAT shells. To the best of the authors’ knowledge, only a couple of works have been published about this topic, specifically, the works by Tornabene et al. [27] and Sarvestani et al. [28]. The former [27] used a similar approach to the one proposed in this paper, whilst the latter [28] adopted a semi-analytical methodology to perform hygro-thermo-mechanical analysis on thin to relatively-thick VAT composite panels. To circumvent that research absence, this paper aims to present additional stress benchmarks for future comparisons. In this manner, the main objective of this manuscript is to accurately predict the 3D linear stress state of tow-steered composite shells and provide stress benchmarks.

The proposed methodology relies on the CUF [29]. In CUF, the accuracy of the model can be fine-tuned straightforwardly since the order of the structural model is treated as input of the analysis. CUF has been adopted to obtain 2D theories, which have been then extended to the analysis of multilayered, composite plates and shells, and later for beam models [30,31,32]. In recent years, CUF has also been employed in the analysis of VAT composites. For instance, Demasi et al. [33] showed some numerical assessments on the stress distribution of VAT plates employing Equivalent Single Layer (ESL) and Layerwise (LW) theories. The vibrations and buckling of variable stiffness plates were studied by Vescovini and Dozio [34] by coupling the CUF and Ritz method. Viglietti et al. [35] introduced 1D elements for the free vibration study of VAT laminates. Lately, Pagani and Sanchez-Majano [36,37] and Sanchez-Majano et al. [38] analysed the influence of manufacturing defects on the mechanical performance of VAT composites.

This manuscript is subdivided as follows: (i) first, a description of the 2D CUF modelling approach for shells is made in Section 2, including a description of VAT over curved domains; then, Section 3 presents the numerical results obtained with CUF shell models. Finally, conclusions are drawn in Section 4.

## 2. Unified Finite Elements for VAT Shells

### 2.1. Preliminary Considerations

**Figure 1:**
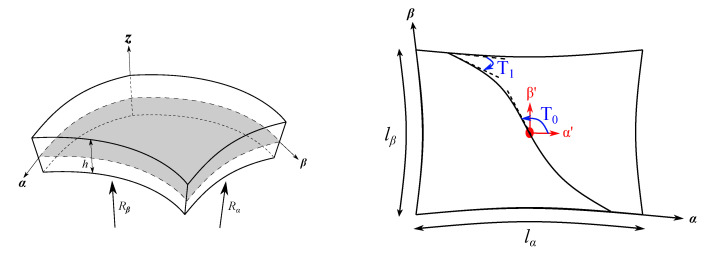
Geometry and reference system of a generic VAT shell model.

In this work, the structures are modelled using refined shell models. A shell is a 2D structural element where the thickness is negligible compared to the other dimensions. Typically, this geometry is described employing an orthogonal curvilinear reference system (α,β,z), as reported in Figure 1, in which α and β indicate the in-plane surface and *z* the thickness direction. For the sake of brevity, the complete description of the shell formulation in the CUF domain is not within the scope of this article. The reader is referred to [32,39].

The transposed displacement, strain and stress vectors for each layer *k* are written as follows:

(1) $$\begin{array}{c} u^{k}={\left\{\;u_{\alpha }^{k},\;u_{\beta }^{k},\;u_{z}^{k}\;\right\}}^{T}\\ \epsilon^{k}={\left\{\epsilon_{\alpha \alpha }^{k},\;\epsilon_{\beta \beta }^{k},\;\epsilon_{zz}^{k},\;\epsilon_{\alpha z}^{k},\;\epsilon_{\beta z}^{k},\;\epsilon_{\alpha \beta }^{k}\right\}}^{T}\\ \sigma^{k}={\left\{\sigma_{\alpha \alpha }^{k},\;\sigma_{\beta \beta }^{k},\;\sigma_{zz}^{k},\;\sigma_{\alpha z}^{k},\;\sigma_{\beta z}^{k},\;\sigma_{\alpha \beta }^{k}\right\}}^{T} \end{array}$$

The displacement-strain relations are written as:

(2) $$\epsilon^{k}=Du^{k}$$

in which D represents the differential operator containing the geometrical relations between strains and displacements. This operator reads as:

(3) $$D=\left[\begin{array}{ccc} \frac{\partial_{\alpha }}{H_{\alpha }} & 0 & \frac{1}{H_{\alpha }R_{\alpha }}\\ 0 & \frac{\partial_{\beta }}{H_{\beta }} & \frac{1}{H_{\beta }R_{\beta }}\\ 0 & 0 & \partial_{z}\\ \partial_{z}-\frac{1}{H_{\alpha }R_{\alpha }} & 0 & \frac{\partial_{\alpha }}{H_{\alpha }}\\ 0 & \partial_{z}-\frac{1}{H_{\beta }R_{\beta }} & \frac{\partial_{\beta }}{H_{\beta }}\\ \frac{\partial_{\beta }}{H_{\beta }} & \frac{\partial_{\alpha }}{H_{\alpha }} & 0 \end{array}\right]$$

where $\partial_{\alpha }=\partial \left(\cdot \right)/\partial_{\alpha }$, $\partial_{\beta }=\partial \left(\cdot \right)/\partial_{\beta }$, $\partial_{z}=\partial \left(\cdot \right)/\partial_{z}$, and $H_{\alpha }$, $H_{\beta }$ are defined as:

(4) $$H_{\alpha }^{k}=A^{k}\left(1+z_{k}/R_{\alpha }^{k}\right),\;\;H_{\beta }^{k}=B^{k}\left(1+z_{k}/R_{\beta }^{k}\right)$$

where $R_{\alpha }^{k}$ and $R_{\beta }^{k}$ denote the radii of the middle surface of the $k^{th}$ layer, and $A^{k}$ and $B^{k}$ indicate the Lamé parameters. Using the constitutive equations, stresses are evaluated as:

(5) $$\sigma^{k}=C^{k}\epsilon^{k}$$

where $C^{k}$ is the material elastic matrix [40,41]. Since in this work VAT structures are investigated, the fibres have a general orientation function of the space coordinates, i.e., θ(α,β). Thus, we write:

(6) $$\sigma^{k}={\tilde{C}}^{k}\epsilon^{k}$$

in which:

(7) $${\tilde{C}}^{k}=T^{T}C^{k}T$$

where T represents the rotation matrix [42]. The matrix ${\tilde{C}}^{k}$ changes pointwise in VAT composite structures.

**Figure 2:**
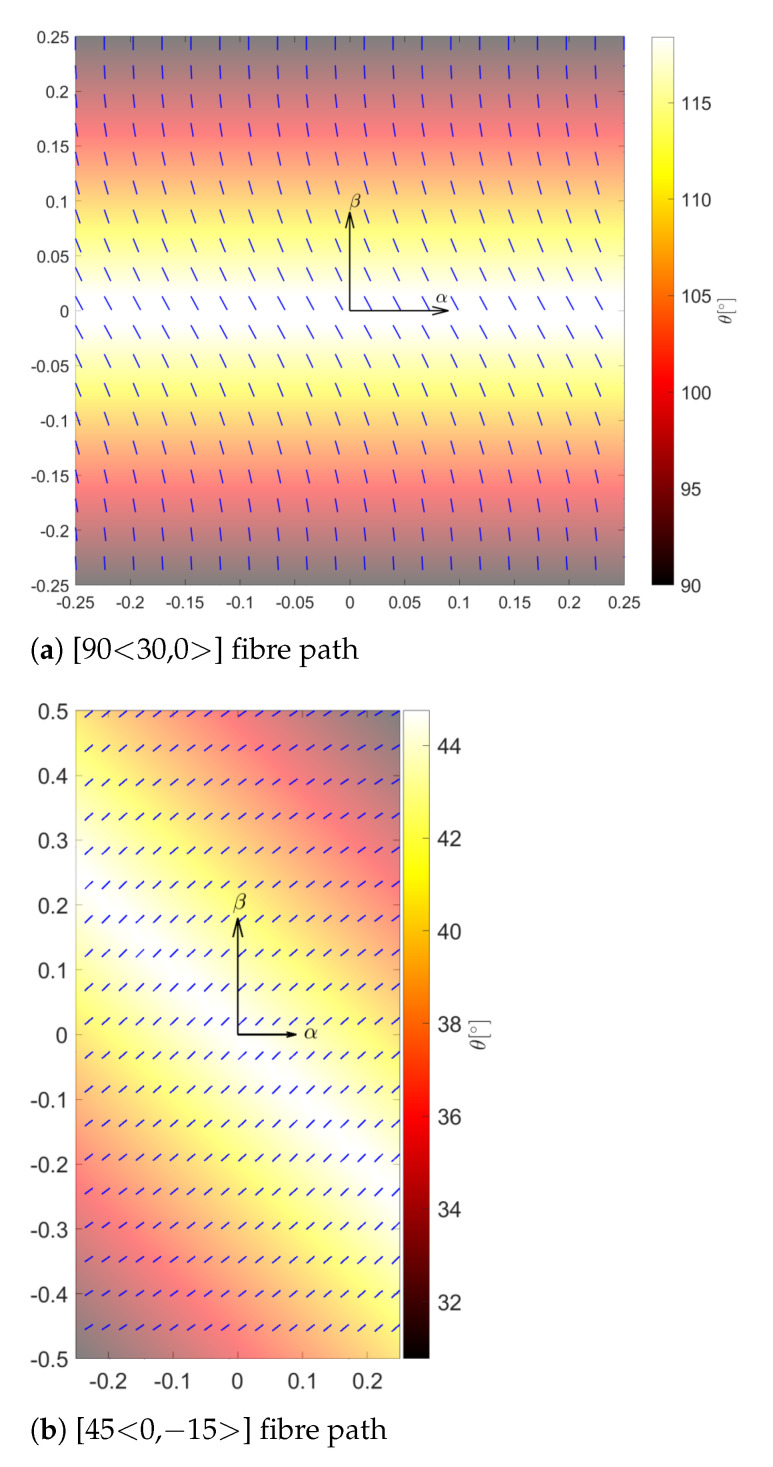
Graphical representation of a spatially varying fibre path over (**a**) squared and (**b**) rectangular domains.

In a VAT structure, the fibre can change continuously along a curvilinear path in each ply. In this way, the laminate has a different stiffness value at each position. A linear fibre angle variation over the lamina is employed in this work, see Figure 1, and the fibre orientation, using the notation of G$\ddot{u}$rdal [43], is formulated as follows:

(8) $$\theta \left(\alpha^{\prime }\right)=\Phi +T_{0}+\frac{\left(T_{1}-T_{0}\right)}{d}\left|\alpha^{\prime }\right|$$

where the fibre path exhibits a rotation of an angle Φ with respect to a certain reference direction. The fibre orientation angle at this point is $T_{0}$ and varies along a direction $\alpha^{\prime }$ oriented by angle Φ from the original coordinate axis α. The fibre orientation reaches the value $T_{1}$ at a characteristic distance *d* from the reference point. By considering this rotation angle, the fibre orientation path θ(α,β) is expressed as $\theta (\alpha^{\prime })$, in which $\alpha^{\prime }=\alpha cos\Phi +\beta sin\Phi$. The parameter *d* is equal to a/2 or b/2 when Φ= 0° or Φ= 90°, where *a* and *b* are the width and length of the 2D structure, respectively. For a better understanding of the fibre path variation along the in-plane, two of the fibre paths considered in the upcoming assessments are represented in Figure 2.

For clarity, unlike commercial codes where the lamination angle is considered constant over the entire element, in the presented methodology, the material coefficients of the VAT structure are evaluated in specific Gauss points [35]. The use of the Gauss point integration technique guarantees a more accurate and efficient analysis of composite VAT structures, since the variability in the material stiffness coefficients $C_{ijkl}$ is accounted in several points for the same FE. Moreover, in the present model, the number of Gauss points per element can be increased independently of the FE type, and thus, it influences the number of degrees of freedom.

### 2.2. Kinematic Assumption, Governing Equation and FE Approximation

In this article, VAT composite shells are modelled employing refined 2D CUF models. In the CUF framework, the refinement of the theory is assumed as an input of the analysis, so low- to higher-order models can be built with ease and in a unified manner (i.e., no ad-hoc formulations are needed to obtain any model). The 3D displacement field is formulated as an arbitrary through-the-thickness expansion of the in-plane variables.

(9) $$u^{k}\left(\alpha ,\beta ,z\right)=F_{\tau }^{k}\left(z\right)u_{\tau }^{k}\left(\alpha ,\beta \right),\;\tau =1,\ldots ,M$$

in which $F_{\tau }$ represents a set of thickness expansion functions, $u_{\tau }$ is the generalised displacement vector depending on the in-plane coordinates α and β, *M* indicates the order of expansion in the thickness direction and the repeated index τ denotes summation. The choice of $F_{\tau }$ is arbitrary and determines the class of the considered 2D CUF shell model. For the sake of brevity, the reader is referred to [29] for a detailed description of the shell theories within the CUF domain.

**Figure 3:**
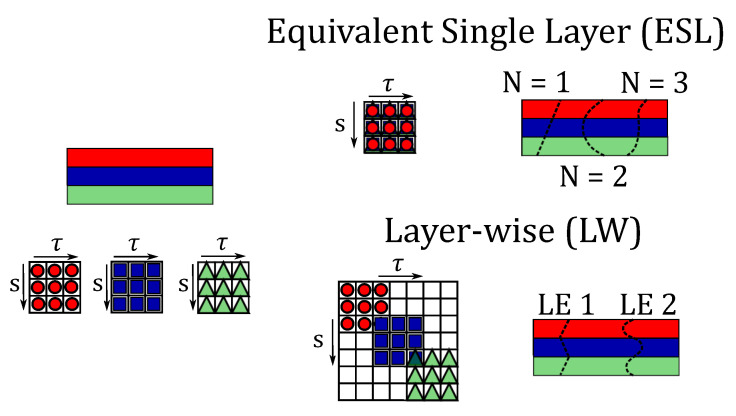
Assembling procedure of the stiffness matrix and behaviour of the primary variables along the thickness of the structure.

The use of laminated composite structures leads to important challenges in the design process. One of the most important assessments is undoubtedly the correct prediction of the stress distribution within the structure. In the literature, ESL and LW theories are typically employed when dealing with composite materials. In this work, both the ESL based on Taylor Expansion (TE) and LW adopting the Lagrange Expansion (LE) are considered. The acronym LDN, used in the following figures, denotes the LE of order N assumed in the *z* direction. The differences in the assembly procedure for ESL and LW and the behaviour of the primary variables along the thickness of the shell are reported in Figure 3.

In detail, in ESL the stiffness matrix is evaluated with the homogenisation technique of the properties of each layer by summing the contributions of each layer. The result is a multilayer configuration modelled as a single layer having a set of variables assumed for the entire cross-section. On the contrary, LW theories divide and expand the displacement field within each material layer. By doing so, homogenisation is carried out at the interface layer. ESL theories exhibit accurate results of the global response (fundamental vibration frequency, transverse deflection), but they are often inaccurate for the 3D stress distributions compared to the LW methodology.

Independently of the selected shell model kinematics, the finite element method (FEM) is used to approximate the in-plane generalised displacement vector employing the Lagrange shape functions $N_{i}\left(\alpha ,\beta \right)$.

(10) $$u_{\tau }^{k}\left(\alpha ,\beta \right)=N_{i}\left(\alpha ,\beta \right)q_{\tau i}^{k},\;i=1,2,\ldots ,N_{n}$$

in which $q_{\tau i}$ denotes the unknown nodal variables, $N_{n}$ indicates the number of nodes per element and *i* stands for summation. For completeness, the classical 2D nine-node quadratic finite element (FE) (Q9) is considered in the following analyses for the shape function in the α,β plane.

The principle of virtual displacements (PVD) is used to derive the expression for the stiffness matrix. PVD states that:

(11) $$\delta L_{int}=\delta L_{ext}$$

in which $\delta L_{int}$ represents the virtual variation of the internal strain energy

(12) $$\delta L_{int}=\int_{V}\delta \epsilon^{T}\sigma dV$$

and $\delta L_{ext}$ is the virtual work of external loading

(13) $$\delta L_{ext}=\delta q_{sj}^{T}p_{sj}+\int_{S}\delta q_{sj}^{T}f_{sj}dS$$

where $p_{sj}$ is the vector of the applied point load components (3 × 1) and $f_{sj}$ is a surface force.

By using Equations (10) and (12), the constitutive law (Equation (6)) and the geometrical relations which results in the following equation for the stiffness matrix:

(14) $$\delta L_{int}=\delta q_{sj}^{T}k^{ij\tau s}q_{\tau i}$$

where $k^{ij\tau s}$ is the 3 × 3 fundamental nucleus (FN), see the CUF book [29] for its derivation. The mathematical expression for FN results in:

(15) $$k^{ij\tau s}=\int_{V}D^{T}\left(N_{i}F_{\tau }\right)\tilde{C}D\left(N_{j}F_{s}\right)dV$$

Conversely, from other CUF-based works, Equation (15) cannot be split into separate integrals where the FE solution and CUF expansion are independently evaluated, and, therefore, a 3D integration is needed. Finally, the global assembled stiffness matrix K is obtained by looping through the indices *i*, *j*, τ, *s*.

## 3. Numerical Results

The stress analyses of VAT shell structures subjected to different loadings are discussed in this section. In particular, flat and curved panels with different material properties, lamination schemes, curvature ratios and boundary conditions were investigated. For this purpose, both the layerwise theory and equivalent single layer approaches are adopted and compared, showing the need to adopt layerwise when evaluating transverse shear stresses. The material properties considered in the following studies are reported in Table 1. These materials were selected from existing literature concerning shell-like structures [32,44].

### 3.1. Simply Supported VAT Flat Panel

**Figure 4:**
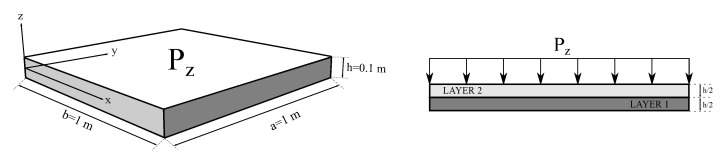
Geometry and loading condition of the simply supported VAT flat panel. The exerted pressure is $P_{z}=10$ kPa.

The first numerical assessment consists of a laminated squared flat panel composed of two layers. This flat panel is simply supported on its four edges, and the stacking sequence is: θ=[0<90,45>,0<0,45>]. A graphite/epoxy composite, whose elastic properties are available in Table 1, is employed in this structure. A graphical representation of the flat panel subjected to a uniform pressure ($P_{z}=10$ kPa) exerted on the top surface, including its dimensions, is illustrated in Figure 4.

**Figure 5:**
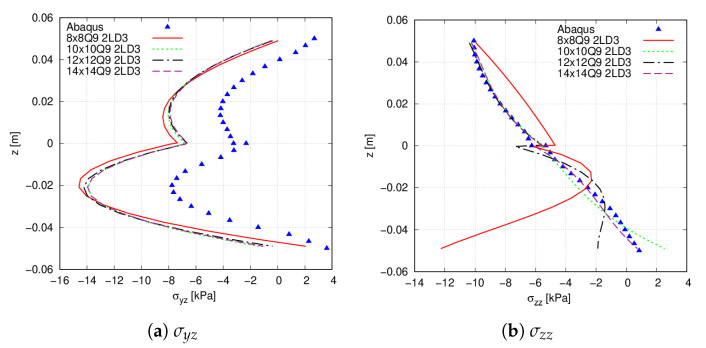
Through-the-thickness stress field, measured at point Q, of the simply supported [0<90,45>,0<0,45>] flat panel. (**a**) $\sigma_{yz}$; (**b**) $\sigma_{zz}$.

First, a convergence study on the in-plane finite element mesh is carried out. The reference results were obtained using commercial software Abaqus [45], where a mesh employing 80×80×16 solid C3D8R elements was utilised. Figure 5 shows the transverse normal ($\sigma_{zz}$) and ($\sigma_{yz}$) shear stresses for different 2D plate models, from 64 Q9 FEs up to 196 Q9 FEs, whereas 2LD3 elements were placed through the thickness direction. Table 2 gathers the six stress components evaluated at point Q (*x* = 0.25 m, *y* = 0.25 m) and z=0.02 m for the different mesh approximations. These results suggest that a 10×10 Q9 mesh approximation provides an accurate evaluation of the stresses. However, by looking at Figure 5b, the lower part of the stress distribution does not match properly with the reference results. A better prediction is provided in this case by the 14×14 mesh approximation. Then, it is appreciated in both Figure 5a and Table 2 that the present approach does not predict the transverse shear stress distribution of the commercial software. These differences are mentioned below. 

**Figure 6:**
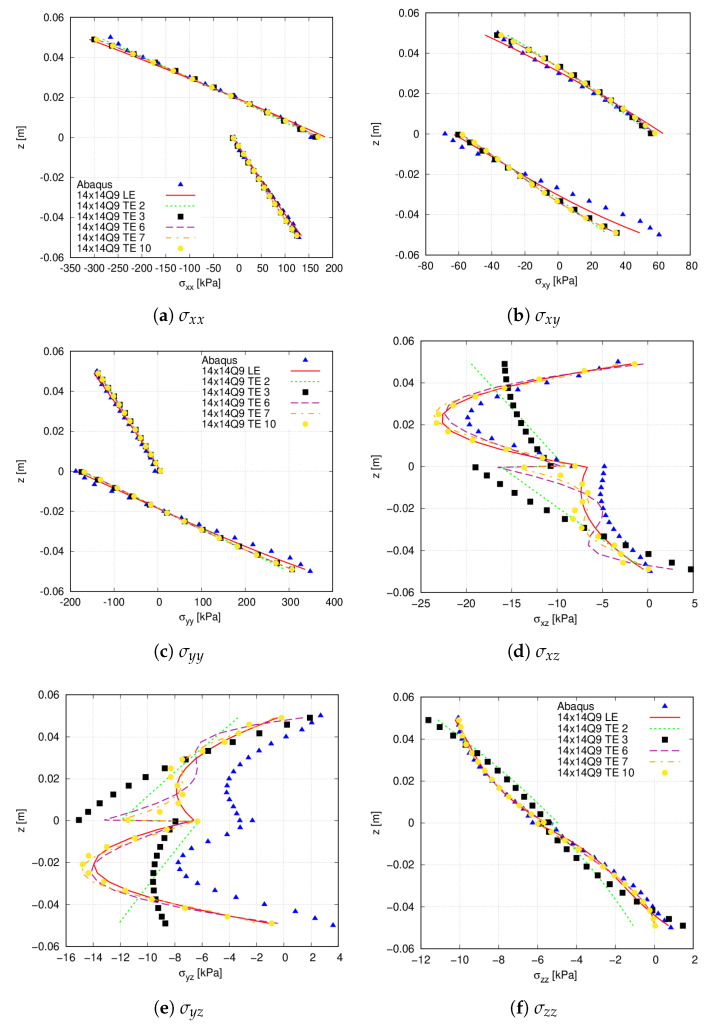
Through-the-thickness stress field, measured at point Q, of the simply supported [0<90,45>,0<0,45>] flat panel. (**a**) $\sigma_{xx}$; (**b**) $\sigma_{xy}$; (**c**) $\sigma_{yy}$; (**d**) $\sigma_{xz}$; (**e**) $\sigma_{yz}$; (**f**) $\sigma_{zz}$. Legend in (**a**,**c**,**f**) apply for all figures in the panel.

To perform an accurate stress prediction, different expansion functions are used for the thickness discretisation. Both LE and TE functions are considered in this study. The through-the-thickness stress distribution is shown in Figure 6. The corresponding stress values are enlisted in Table 3 for different 2D theories. As appreciated, the LW approach provides the more accurate stress results when 2LD3 theories are employed. The outcomes suggest that the ESL model is sufficient to evaluate the in-plane normal and shear stresses, whilst their accuracy diminishes when predicting the transverse shear stresses. Concerning the latter, TE 6, TE 7 and TE 10 provide a similar distribution compared to 2LD3 but present equal or higher DOF. However, oscillations appear close to the interface for those two theories. This means that even considering up to tenth-order terms, an accurate evaluation of these stress components is not available. Additionally, there exist differences between the Abaqus model and the 2LD3 relative to shear transverse stresses, especially for the $\sigma_{yz}$ component. These are due to the different formulations that both models present: the dedicated Abaqus model uses a solid 3D linear model (C3D8R element), whereas a LW formulation is achieved with the present approach. Indeed, Abaqus does not respect the stress-free condition at the plate’s bottom and top as shown in Figure 6e.

### 3.2. Clamped VAT Curved Panel

**Figure 7:**
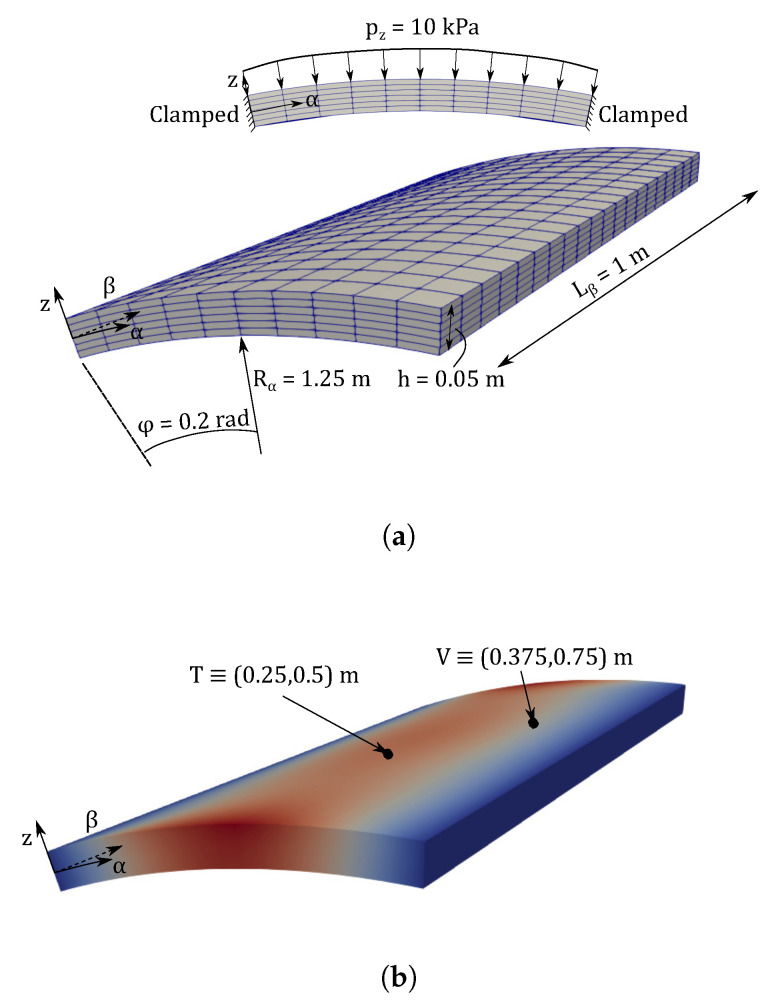
Graphical description of the clamped curved VAT panel: (**a**) geometry and boundary conditions; (**b**) points where magnitudes are measured.

The second numerical assessment considers a curved VAT panel. The structure has one meter length, an opening angle φ=0.2 rad, internal radius $R_{\alpha }=1.25$ m and thickness h=0.05 m. The panel is clamped on its longitudinal edges and an uniform pressure $p_{z}=10$ kPa is exerted on the upper surface, as depicted in Figure 7. The stacking sequence is chosen to be $\theta ={\left[0<0,50>,90<0,75>,45<0,15>\right]}_{s}$. The material properties are gathered in Table 1.

**Figure 8:**
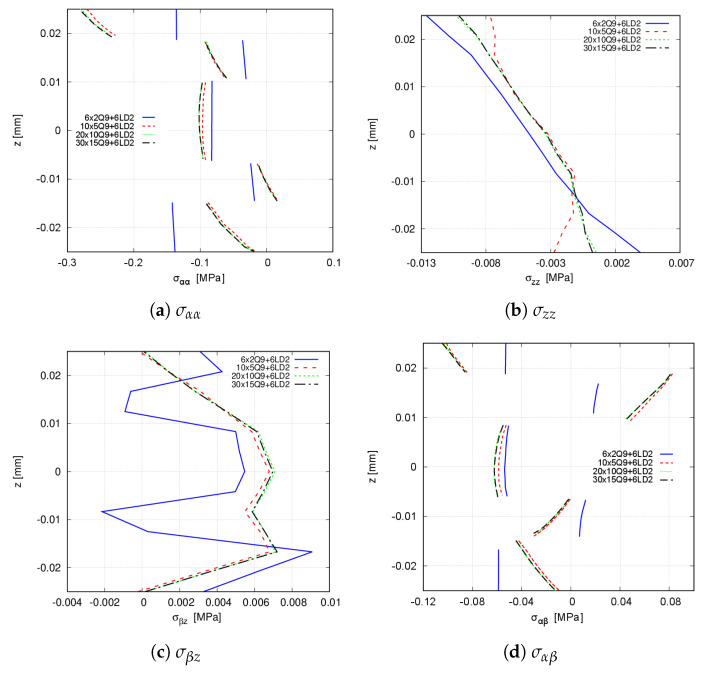
Convergence of the through-the-thickness stress distribution for the ${\left[0<0,50>,90<0,75>,45<0,15>\right]}_{s}$ clamped VAT shell calculated at point V. CUF models. (**a**) $\sigma_{\alpha \alpha }$; (**b**) $\sigma_{zz}$; (**c**) $\sigma_{\beta z}$; (**d**) $\sigma_{\alpha \beta }$.

**Figure 9:**
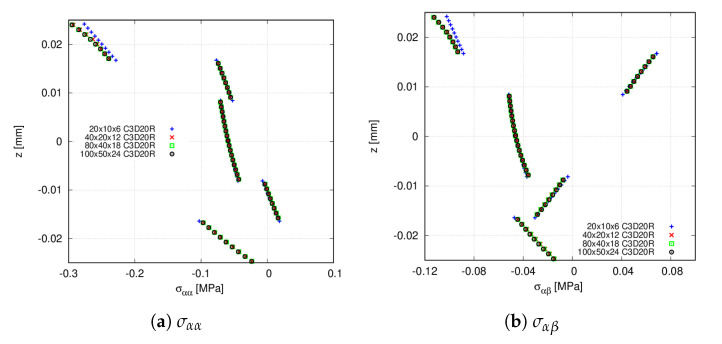
Convergence of the through-the-thickness stress distribution for the ${\left[0<0,50>,90<0,75>,45<0,15>\right]}_{s}$ clamped VAT shell calculated at point V. Abaqus quadratic models. (**a**) $\sigma_{\alpha \alpha }$; (**b**) $\sigma_{\alpha \beta }$.

First, a mesh convergence analysis is performed by varying the number of in-plane finite elements, while a 6LD2 expansion theory is employed in the thickness direction. Table 4 and Table 5 show the convergence of the transverse displacement and the stresses computed at points T and V, respectively. Figure 8 shows the stress distribution at point V using the present approach, whilst the convergence of the Abaqus model is available in Figure 9. Small differences between FE meshes are appreciated when accounting for the transverse displacement and the in-plane stresses $\sigma_{\alpha \alpha }$ and $\sigma_{\alpha \beta }$. However, these differences are more evident for the transverse stresses $\sigma_{zz}$ and $\sigma_{\beta z}$. From these results, it is inferred that a 20 × 10Q9 + 6LD2 mesh approximation thoroughly predicts the stress distribution.

**Figure 10:**
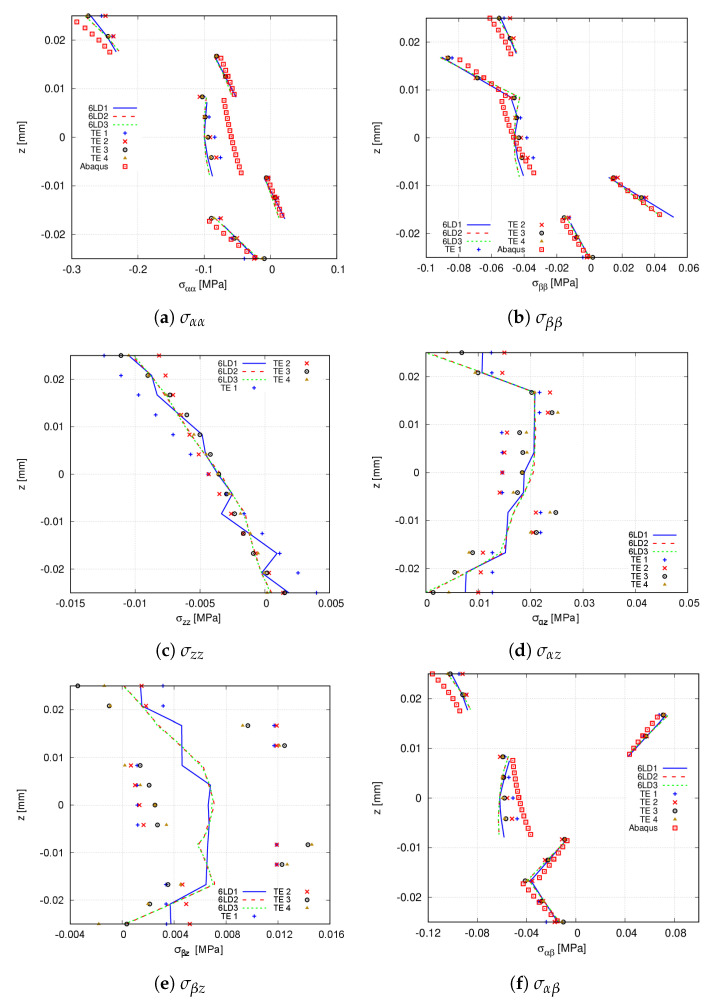
Through-the-thickness stress distribution of the ${\left[0<0,50>,90<0,75>,45<0,15>\right]}_{s}$ clamped VAT shell calculated at point V for the different expansion theories. The Abaqus solid model comprises 80 × 40 × 18 C3D20R quadratic elements. (**a**) $\sigma_{\alpha \alpha }$; (**b**) $\sigma_{\beta \beta }$; (**c**) $\sigma_{zz}$; (**d**) $\sigma_{\alpha z}$; (**e**) $\sigma_{\beta z}$; (**f**) $\sigma_{\alpha \beta }$.

In the following, stress distributions provided by high-order LE and TE expansion theories are compared with commercial software Abaqus. For such purpose, the 20 × 10Q9 FE mesh approximation is employed. The Abaqus solid model utilises 80 × 40 × 18 C3D20R quadratic elements. Table 6 and Figure 10 provide the stress components computed at point V of the structure. From the former, it is inferred that TE theories are unable to predict the shear stress $\sigma_{\beta z}$, while for the remaining stress components, these expansions are in agreement with 6LD2 and 6LD3. The latter demonstrates that 6LD1 is not capable of retrieving response for any stress component. It is also shown that TE models provide an accurate evaluation of the in-plane terms while presenting difficulties when predicting the transverse components, especially the shear stresses $\sigma_{\alpha z}$ and $\sigma_{\beta z}$. The Abaqus prediction of the transverse shear stresses are not reported in Figure 10c–e since it is not feasible to obtain accurate results for such stress components.

### 3.3. Hinged VAT Shell

**Figure 11:**
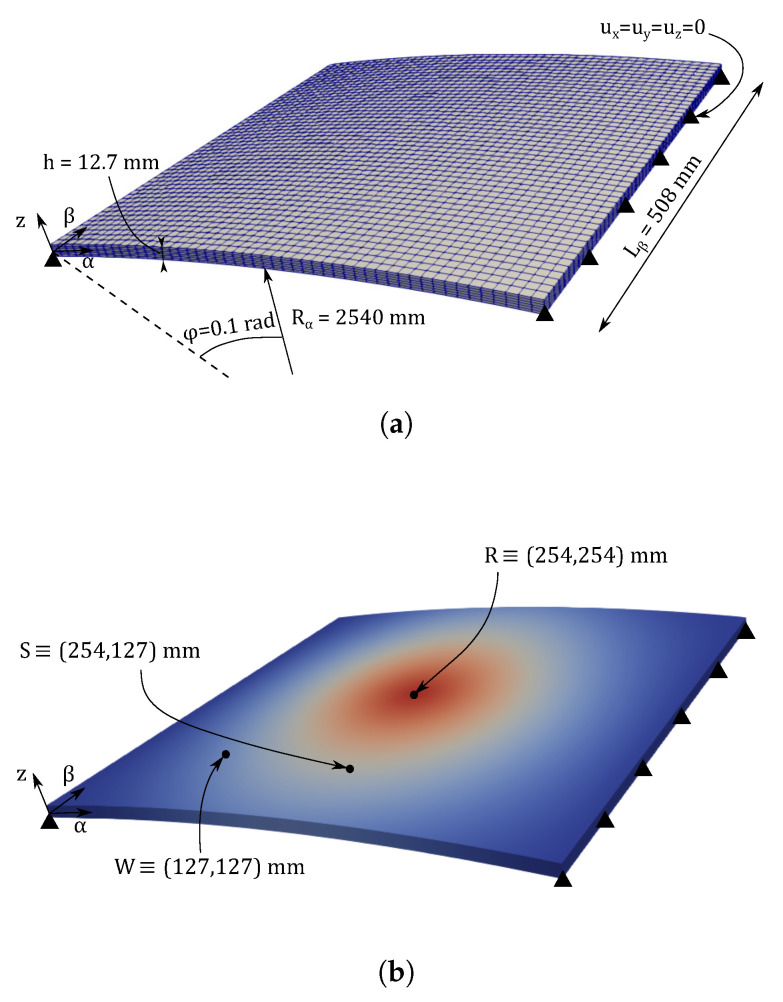
Graphical description of the hinged VAT shell: (**a**) geometry and boundary conditions; (**b**) points where magnitudes are measured.

The third numerical assessment consists of a hinged VAT shell structure. This curved panel comprises three composite laminae where the fibres are steered following the stacking sequence θ=[90<30,0>,0<30,0>,90<30,0>]. A graphical representation of the VAT shell is available in Figure 11, and the material properties are enlisted in Table 1.

Specifically, a convergence analysis is first conducted to find the mesh that provides the most accurate results; then, a comparison between ESL and LW models is carried out. Finally, the effect of the fibre orientation parameters $T_{0}$ and $T_{1}$ on the stress distribution is addressed.

#### 3.3.1. Mesh Convergence Analysis

**Figure 12:**
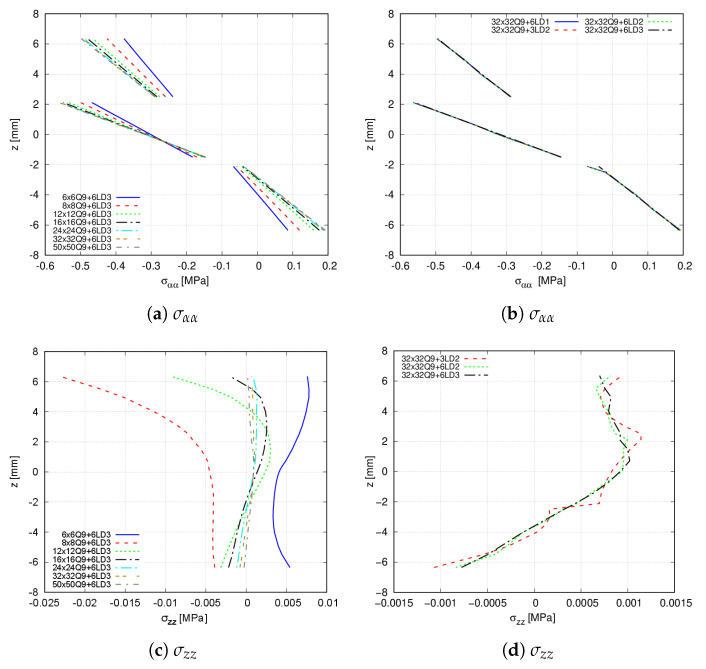
Through-the-thickness stress distribution of the [90<30,0>,0<30,0>,90<30,0>] hinged VAT shell calculated at point R. (**a**) $\sigma_{\alpha \alpha }$; (**b**) $\sigma_{\alpha \alpha }$; (**c**) $\sigma_{zz}$; (**d**) $\sigma_{zz}$.

A convergence study concerning the number of in-plane finite elements is conducted. Likewise, the through-the-thickness expansion order is addressed. Table 7 and Table 8 show the convergence results for both the transverse displacement and the stress distribution at point R and z=6.35 mm, and point S, respectively. Then, in Figure 12, it is appreciated that a 32 × 32 Q9 mesh approximation is necessary to retrieve an accurate evaluation of both the transverse displacement and the stress distributions. The effect of the different expansion functions on the latter is also accounted for in the right panel of Figure 12, especially in the out-of-plane stress variation. Indeed, it is demonstrated that a 6LD2 expansion is required to predict accurately such distributions.

#### 3.3.2. ESL and LW Theories for the Analysis of Laminated VAT Shells

**Figure 13:**
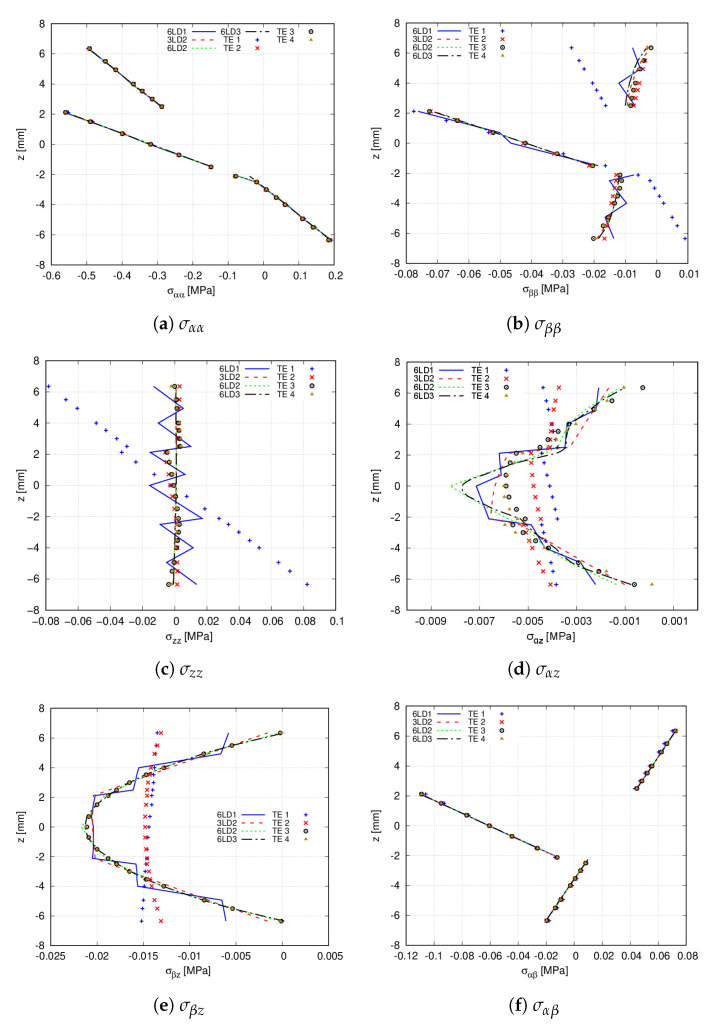
Through-the-thickness stress distribution of the [90<30,0>,0<30,0>,90<30,0>] hinged VAT shell calculated at point R for the different expansion theories. (**a**) $\sigma_{\alpha \alpha }$; (**b**) $\sigma_{\beta \beta }$; (**c**) $\sigma_{zz}$; (**d**) $\sigma_{\alpha z}$; (**e**) $\sigma_{\beta z}$; (**f**) $\sigma_{\alpha \beta }$.

Then, a comparison between TE and LE expansion functions is carried out. Table 9 enlists the stress values at point R and z=0 mm for the different expansion theories. Subsequently, Figure 13 shows the stress variation provided by these expansions. It is inferred that TE 2, TE 3 and TE 4 theories are able to predict the same in-plane stress distribution that high-order LD theories provide with a fraction of DOF. However, differences appear in the evaluation of the transverse stress components. For instance, TE 1 is not enough to catch the transverse normal stress distribution. Then, as appreciated in Figure 13d,e, TE 1 and TE 2 do not predict the trend of the transverse shear stresses. Moreover, TE 3 and TE 4 present difficulties when retrieving $\sigma_{\alpha z}$, as depicted in Figure 13d.

#### 3.3.3. Effect of the Fibre Orientations on the Stress Distribution

**Figure 14:**
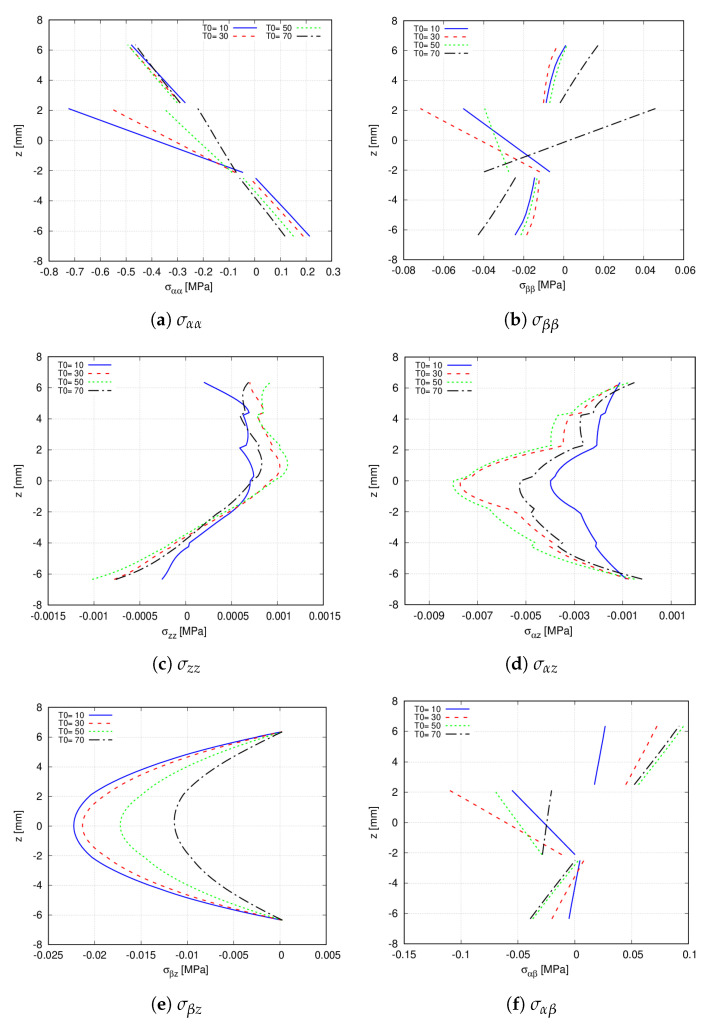
Effect of $T_{0}$ in the through-the-thickness distribution at point S of the $\left[90<T_{0},0>,0<T_{0},0>,90<T_{0},0>\right]$ hinged VAT shell. A 32 × 32Q9 + 6LD3 mesh approximation is used for each analysis. (**a**) $\sigma_{\alpha \alpha }$; (**b**) $\sigma_{\beta \beta }$; (**c**) $\sigma_{zz}$; (**d**) $\sigma_{\alpha z}$; (**e**) $\sigma_{\beta z}$; (**f**) $\sigma_{\alpha \beta }$.

First, the effect of $T_{0}$ on the stress distribution, evaluated at point R, is addressed. Precisely, $T_{0}$ takes the following values $T_{0}={\left[10,30,50,70\right]}^{\circ }$. For these analyses, the 32 × 32 Q9 mesh approximation coupled with the 6LD3 expansion theory in the thickness direction is employed. Figure 14 illustrates the influence of $T_{0}$ on the stress distribution. On the one hand, it is appreciated that the magnitude of the transverse stresses is affected by $T_{0}$. Nevertheless, a sign variation of the stress component, due to $T_{0}$, is not foreseen. On the other hand, concerning the in-plane stresses, $T_{0}$ varies its magnitude and the stress sign. That is, in $\sigma_{\beta \beta }$, the second layer is subjected to compressive stresses when $T_{0}={\left[10,30,50\right]}^{\circ }$ and to tension stresses when $T_{0}=70^{\circ }$.

**Figure 15:**
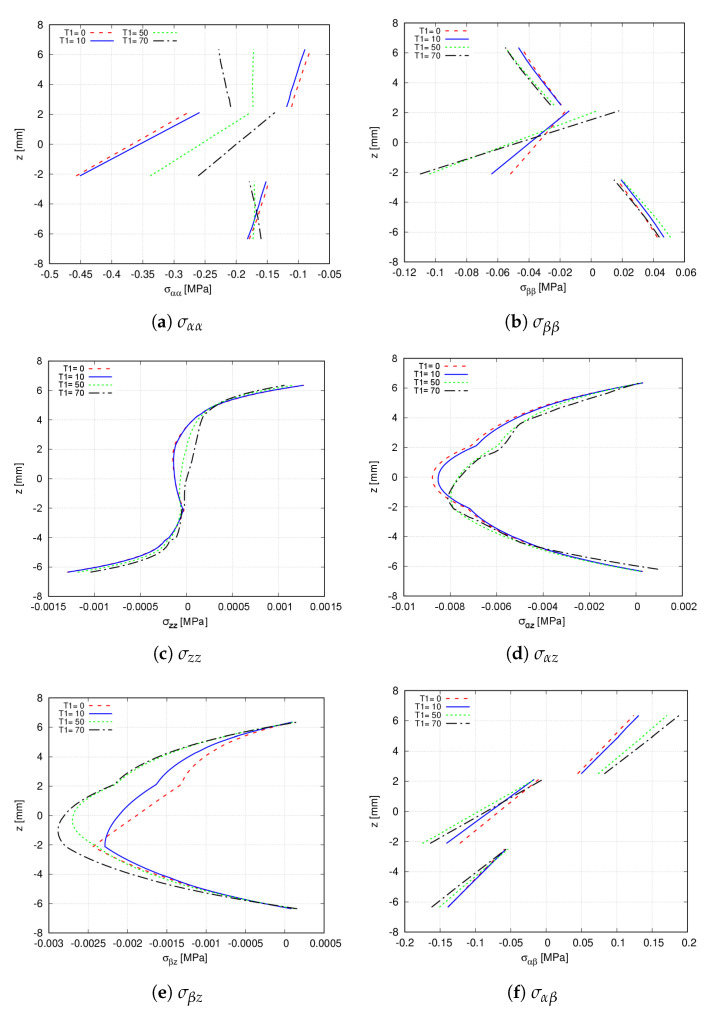
Effect of $T_{1}$ in the through-the-thickness distribution at point W of the $\left[90<30,T_{1}>,0<30,T_{1}>,90<30,T_{1}>\right]$ hinged VAT shell. A 32 × 32Q9 + 6LD3 mesh approximation is used for each analysis. (**a**) $\sigma_{\alpha \alpha }$; (**b**) $\sigma_{\beta \beta }$; (**c**) $\sigma_{zz}$; (**d**) $\sigma_{\alpha z}$; (**e**) $\sigma_{\beta z}$; (**f**) $\sigma_{\alpha \beta }$.

To study how the variation of $T_{1}$ influences the stresses over the VAT shell, the initial [90<30,0>,0<30,0>,90<30,0>] stacking sequence becomes $\left[90<30,T_{1}>,0<30,T_{1}>,90<30,T_{1}>\right]$, where $T_{1}={\left[0,10,50,70\right]}^{\circ }$. The stress components are evaluated at point W, where the effect of $T_{1}$ is appreciated. Figure 15 illustrates the through-the-thickness stress distribution. It is observed that the transverse stresses are not greatly affected by the fibre angle variation. On the contrary, the in-plane components show evident changes, especially the normal stresses where differences are more obvious in the middle layer.

## 4. Concluding Remarks

This paper has presented high-order finite elements to accurately evaluate linear static stresses over variable angle tow (VAT) shell structures. The Carrera Unified Formulation (CUF) constitutes the framework in which such numerical models are embedded because of its capabilities for achieving low fidelity to high-order structural models in a systematic manner. No approximations have been made on the shell geometry nor on the strains. Several geometries, boundary and loading conditions, spatially varying fibre paths and materials have been analysed and compared to commercial software Abaqus. The results suggest that:

- Equivalent Single Layer (ESL) and Layerwise (LW) are in good agreement with commercial software formulations for the analysis of flat panels;
- In the case of curved panels, Abaqus and ESL provide similar results for the in-plane stress prediction when compared to LW models. Nevertheless, obvious differences arise in the evaluation of the transverse stresses. Such differences can be mitigated by employing high-order TE;
- The selection of the fibre path parameters ($\phi ,T_{0},T_{1}$) can be fine-tuned to conveniently alter the stress distribution at certain points of interest of the structure.

Future works will concern the linear and nonlinear behaviour of sandwich-like VAT shells and applications for structural failures, such as postbuckling.

## Figures and Tables

**Table 1:** Elastic properties of the materials used for the different analysed VAT structures.

Case	$E_{1}$ [GPa]	$E_{2}=E_{3}$ [GPa]	$G_{12}$ [GPa]	$G_{23}$ [GPa]	$\nu_{12}$ [-]	$\nu_{23}$ [-]
Simply supported VAT flat panel	143.17	9.64	6.09	3.12	0.252	0.349
Clamped VAT curved panel	165.0	9.0	5.60	2.80	0.34	0.50
Hinged VAT shell	3.30	1.10	0.66	0.66	0.25	0.25

**Table 2:** Stress state of the simply supported [0<90,45>,0<0,45>] flat panel evaluated at point Q and z=0.02 m for different FE mesh approximations. Each discretization employs 2LD3 elements through the thickness direction.

Model	DOF	$\sigma_{\mathrm{xx}}$ [kPa]	$\sigma_{\mathrm{yy}}$ [kPa]	$\sigma_{\mathrm{zz}}$ [kPa]	$\sigma_{\mathrm{xz}}$ [kPa]	$\sigma_{\mathrm{yz}}$ [kPa]	$\sigma_{\mathrm{xy}}$ [kPa]
Abaqus	334,611	−11.13	−56.06	−8.33	−19.64	−4.02	21.64
8×8 Q9	6069	−4.19	−53.26	−6.67	−21.68	−7.95	27.37
10×10 Q9	9261	−12.75	−54.96	−8.35	−22.73	−7.77	22.52
12×12 Q9	13,125	−8.33	−54.45	−8.43	−22.10	−7.75	25.03
14×14 Q9	17,661	−12.71	−55.29	−8.39	−22.61	−7.66	22.67

**Table 3:** Point Q stress state of the simply supported [0<90,45>,0<0,45>] flat panel at z=0.02 m for different structural theories and a 14×14 Q9 FE mesh approximation.

Theory	DOF	$\sigma_{\mathrm{xx}}$ [kPa]	$\sigma_{\mathrm{yy}}$ [kPa]	$\sigma_{\mathrm{zz}}$ [kPa]	$\sigma_{\mathrm{xz}}$ [kPa]	$\sigma_{\mathrm{yz}}$ [kPa]	$\sigma_{\mathrm{xy}}$ [kPa]
TE 1	5046	−26.65	−55.07	−24.49	−7.95	−11.68	18.71
TE 2	7569	−17.13	−51.08	−7.33	−13.49	−8.43	22.17
TE 3	10,092	−10.65	−50.57	−7.57	−13.96	−10.15	25.19
TE 4	12,615	−11.79	−51.04	−8.44	−19.43	−6.48	24.99
TE 5	15,138	−12.53	−51.38	−8.37	−20.96	−7.07	24.66
TE 6	17,661	−12.94	−51.49	−8.41	−21.64	−6.48	24.47
TE 7	20,184	−13.06	−51.52	−8.39	−22.97	−7.45	24.44
TE 10	27,753	−13.30	−51.59	−8.35	−23.27	−8.30	24.32
2LD3	17,661	−13.11	−55.35	−8.22	−22.54	−7.63	22.48

**Table 4:** Finite element mesh convergence for the transverse displacement calculated at point T and z=0.025 m for the ${\left[0<0,50>,90<0,75>,45<0,15>\right]}_{s}$ clamped VAT shell.

FE Mesh	DOF	$-u_{z}\cdot 10^{6}$ [m]
6 × 2 Q9 + 6LD2	2535	2.37
10 × 5 Q9 + 6LD2	9009	2.93
20 × 10 Q9 + 6LD2	33,579	3.00
30 × 15 Q9 + 6LD2	73,749	3.01

**Table 5:** Finite element mesh convergence for the stresses calculated at point V and z=0 m for the ${\left[0<0,50>,90<0,75>,45<0,15>\right]}_{s}$ clamped VAT shell.

FE Mesh	DOF	$\sigma_{\alpha \alpha }$ [Pa]	$\sigma_{\mathrm{zz}}$ [Pa]	$\sigma_{\beta z}$ [Pa]	$\sigma_{\alpha \beta }$ [Pa]
6 × 2 Q9 + 6LD2	2535	−8.31 $\times \;10^{4}$	−4.65 $\times \;10^{3}$	5.47 $\times \;10^{3}$	−5.18 $\times \;10^{4}$
10 × 5 Q9 + 6LD2	9009	−9.67 $\times \;10^{4}$	−3.20 $\times \;10^{3}$	6.78 $\times \;10^{3}$	−5.97 $\times \;10^{4}$
20 × 10 Q9 + 6LD2	33,579	−1.00 $\times \;10^{5}$	−3.38 $\times \;10^{3}$	7.08 $\times \;10^{3}$	−6.21 $\times \;10^{4}$
30 × 15 Q9 + 6LD2	73,749	−9.98 $\times \;10^{4}$	−3.32 $\times \;10^{3}$	6.97 $\times \;10^{3}$	−6.17 $\times \;10^{4}$

**Table 6:** Comparison of the in-plane and out-of-plane stresses provided by different expansion theories for the ${\left[0<0,50>,90<0,75>,45<0,15>\right]}_{s}$ clamped VAT shell. Stresses are computed at point V and z=0 using the 20 × 10 Q9 mesh approximation.

Theory	DOF	$\sigma_{\alpha \alpha }$ [Pa]	$\sigma_{\mathrm{zz}}$ [Pa]	$\sigma_{\beta z}$ [Pa]	$\sigma_{\alpha \beta }$ [Pa]
TE 1	5166	−8.43 $\times \;10^{4}$	−4.37 $\times \;10^{3}$	1.18 $\times \;10^{3}$	−5.10 $\times \;10^{4}$
TE 2	7749	−9.11 $\times \;10^{4}$	−4.33 $\times \;10^{3}$	1.33 $\times \;10^{3}$	−5.57 $\times \;10^{4}$
TE 3	10,332	−9.44 $\times \;10^{4}$	−3.55 $\times \;10^{3}$	2.53 $\times \;10^{3}$	−5.80 $\times \;10^{4}$
TE 4	12,915	−9.65 $\times \;10^{4}$	−3.62 $\times \;10^{3}$	2.53 $\times \;10^{3}$	−5.95 $\times \;10^{4}$
6LD1	18,081	−9.99 $\times \;10^{4}$	−3.31 $\times \;10^{3}$	6.62 $\times \;10^{3}$	−6.19 $\times \;10^{4}$
6LD2	33,579	−1.00 $\times \;10^{5}$	−3.38 $\times \;10^{3}$	7.08 $\times \;10^{3}$	−6.21 $\times \;10^{4}$
6LD3	49,077	−1.00 $\times \;10^{5}$	−3.53 $\times \;10^{3}$	6.89 $\times \;10^{3}$	−6.21 $\times \;10^{4}$

**Table 7:** Expansion order and finite element mesh convergence for the transverse displacement calculated at point R and z=6.35 mm for the [90<30,0>,0<30,0>,90<30,0>] hinged VAT shell.

Expansion Order	FE Mesh	DOF	$-u_{z}$ [mm]
6LD1	8 × 8 Q9	6069	0.905
	12 × 12 Q9	13,125	0.938
	16 × 16 Q9	22,869	0.953
	24 × 24 Q9	50,421	0.972
	32 × 32 Q9	88,725	0.985
3LD2	8 × 8 Q9	6069	0.903
	12 × 12 Q9	13,125	0.936
	16 × 16 Q9	22,869	0.951
	24 × 24 Q9	50,421	0.969
	32 × 32 Q9	88,725	0.982
6LD2	8 × 8 Q9	11,271	0.905
	12 × 12 Q9	24,375	0.939
	16 × 16 Q9	42,471	0.955
	24 × 24 Q9	93,639	0.975
	32 × 32 Q9	164,775	0.990
6LD3	8 × 8 Q9	16,473	0.905
	12 × 12 Q9	35,635	0.940
	16 × 16 Q9	62,073	0.955
	24 × 24 Q9	136,857	0.975
	32 × 32 Q9	240,825	0.990
	50 × 50 Q9	581,457	1.020

**Table 8:** Expansion order and finite element mesh convergence for $\sigma_{\alpha \alpha }$, $\sigma_{zz}$, $\sigma_{\beta z}$, and $\sigma_{\alpha \beta }$ at point R and z=0 mm for the [90<30,0>,0<30,0>,90<30,0>] hinged VAT shell.

Expansion Order	FE Mesh	DOF	$\sigma_{\alpha \alpha }$ [MPa]	$\sigma_{\mathrm{zz}}$ [MPa]	$\sigma_{\beta z}$ [MPa]	$\sigma_{\alpha \beta }$ [MPa]
6LD1	8 × 8 Q9	6069	−0.312	−0.0166	−0.0176	−0.0559
	12 × 12 Q9	13,125	−0.317	−0.0133	−0.0207	−0.0591
	16 × 16 Q9	22,869	−0.321	−0.0151	−0.0216	−0.0603
	24 × 24 Q9	50,421	−0.323	−0.0156	−0.0208	−0.0608
	32 × 32 Q9	88,725	−0.324	−0.0156	−0.0204	−0.0610
3LD2	8 × 8 Q9	6069	−0.308	−0.0047	−0.0190	−0.0557
	12 × 12 Q9	13,125	−0.313	−0.0206	−0.0210	−0.0588
	16 × 16 Q9	22,869	−0.316	−0.0121	−0.0216	−0.0599
	24 × 24 Q9	50,421	−0.318	0.00080	−0.0209	−0.0605
	32 × 32 Q9	88,725	−0.319	0.00080	−0.0205	−0.0607
6LD2	8 × 8 Q9	11,271	−0.308	−0.00453	−0.0202	−0.0558
	12 × 12 Q9	24,375	−0.313	−0.00232	−0.0224	−0.0588
	16 × 16 Q9	42,471	−0.316	−0.00130	−0.0229	−0.5997
	24 × 24 Q9	93,639	−0.318	−0.00092	−0.0222	−0.0605
	32 × 32 Q9	164,775	−0.318	−0.00094	−0.0217	−0.0607
6LD3	8 × 8 Q9	16,473	−0.308	−0.00454	−0.0198	−0.0558
	12 × 12 Q9	35,635	−0.313	0.00237	−0.0219	−0.0588
	16 × 16 Q9	62,073	−0.316	0.00137	−0.0225	−0.0599
	24 × 24 Q9	136,857	−0.318	0.00092	−0.0218	−0.0605
	32 × 32 Q9	240,825	−0.318	0.00092	−0.0213	−0.0607
	50 × 50 Q9	581,457	−0.319	0.00088	−0.0202	−0.0608

**Table 9:** Comparison of the in-plane and out-of-plane stresses of the hinged VAT shell provided by the different expansion theories. Stresses are computed at point S and z=0 mm using the 32 × 32 Q9 mesh.

Theory	DOF	$\sigma_{\alpha \alpha }$ [MPa]	$\sigma_{\mathrm{zz}}$ [MPa]	$\sigma_{\beta z}$ [MPa]	$\sigma_{\alpha \beta }$ [MPa]
TE 1	25,350	−0.316	−0.00267	−0.0144	−0.0599
TE 2	38,025	−0.320	−0.00262	−0.0148	−0.0606
TE 3	50,700	−0.319	−0.00053	−0.0211	−0.0606
TE 4	63,375	−0.319	−0.00052	−0.0212	−0.0607
6LD1	88,725	−0.324	−0.01560	−0.0204	−0.0610
3LD2	88,725	−0.319	0.00080	−0.0205	−0.0607
6LD2	164,775	−0.318	−0.00094	−0.0217	−0.0607
6LD3	240,825	−0.318	−0.00094	−0.0217	−0.0607
